# Supplementary material for: Validation of quantitative assessment of indocyanine green fluorescent imaging in a one-vessel model
Source: PLoS One. 2020 Nov 18;15(11):e0240188. doi: 10.1371/journal.pone.0240188 (PMC7673564; doi:10.1371/journal.pone.0240188)
Supplement: S1 File — (PDF) [file pone.0240188.s001.pdf]

Freie und Hansestadt Hamburg
Behörde für Gesundheit und Verbraucherschutz

BGV, Billstraße 80, D - 20539 Hamburg

Universitätsklinikum Hamburg-Eppendorf
Klinik und Poliklinik für Gefäßmedizin
Frau Dr. med. Anna Dupree
Martinistr. 52
20246 Hamburg

Amt für Verbraucherschutz
Lebensmittelsicherheit und Veterinärwesen

Billstraße 80
D - 20539 Hamburg
Telefon 040-428-37-2068
Telefax 040-42837 – 3600

Ansprechpartnerin: Dr. C. Soltau
Zimmer 5.14
E-Mail Christiane.Soltau@bgv.hamburg.de

Gz.: V1305 / 591-00.33

Hamburg, 05.03.2015mko

Nachrichtlich: Dres. Haemisch, Tiemann, **Budack**, Peters

Nr. 113/14
(bei Rückfragen bitte angeben)

Genehmigung zur Durchführung von Versuchen an Wirbeltieren
- Ihr Antrag vom 24.11.2014, hier vollständig eingegangen am 01.12.2014 mit der Bezeichnung „Validierung der Fluoreszenzangiographie zur intraoperativen Beurteilung und Quantifizierung der Mesenterialperfusion"

Sehr geehrte Frau Dr. Dupree,

aufgrund § 8 des Tierschutzgesetzes vom 18. Mai 2006 (BGBl. I S. 1207, 1313), in der derzeit gültigen Fassung, wird Ihnen hiermit unter dem Vorbehalt des jederzeitigen Widerrufs und der nachträglichen Aufnahme von Auflagen die Genehmigung zur Durchführung des nachstehenden Versuchsvorhabens erteilt:

„Validierung der Fluoreszenzangiographie zur intraoperativen Beurteilung und Quantifizierung der Mesenterialperfusion"

Leiter des Versuchsvorhabens:	Frau Dr. Sabine Wipper
Stellvertretender Leiter:	Herr PD. Dr. Oliver Mann
Ort der Durchführung:	UKE
Anzahl und Art der Versuchstiere:	80 Hausschweine
Diese Genehmigung gilt bis zum:	31.03.2018

Für eine rückblickende Bewertung sind Unterlagen gem. § 35 Tierschutz-Versuchstierverordnung (TierSchVersV) nicht einzureichen.

Auflage:
Herr Philipp von Kroge darf aufgrund fehlender Qualifikationsnachweise nicht an dem Versuch mitarbeiten.

Hinweise:

Änderungen des genehmigten Versuchsvorhabens sind unverzüglich anzuzeigen oder zu beantragen (§ 34 Abs. 1 und Abs. 3 TierSchVersV).

Der Wechsel des Leiters des Versuchsvorhabens oder seines Stellvertreters ist unverzüglich anzuzeigen (§ 34 Abs. 2 TierSchVersV).

Bei der Durchführung der Versuchsvorhaben sind die Bestimmungen der TierSchVersV, hier insbesondere §§ 15 bis 25 und §§ 27 bis 30 und die Anforderungen des Tierschutzgesetzes zu beachten.

Gebühren:

Diese Genehmigung ist gebührenpflichtig gemäß § 3 Absatz 1 Gebührengesetz in Verbindung mit Teil V Ziffer 1.1.2 der Anlage zur Gebührenordnung für das öffentliche Gesundheitswesen. Ein Gebührenbescheid wird mit gesonderter Post übersandt.

Rechtsbehelfsbelehrung:

Gegen diesen Bescheid kann innerhalb eines Monats nach Bekanntgabe Widerspruch erhoben werden. Der Widerspruch ist bei der im Briefkopf angegebenen Behörde einzulegen.

Mit freundlichen Grüßen

Dr. Christiane Soltau
