# Supplementary material for: Validation of quantitative assessment of indocyanine green fluorescent imaging in a one-vessel model
Source: PLoS One. 2020 Nov 18;15(11):e0240188. doi: 10.1371/journal.pone.0240188 (PMC7673564; doi:10.1371/journal.pone.0240188)
Supplement: Attachment — Submitted filename: Response to reviewers.docx [file pone.0240188.s002.docx]

Dear Editor, dear reviewers,

Thank you for suggestions concerning our manuscript.

1)The paper requires English correcrion by native speaker before acceptance

The manuscript has been proofed and edited by the Springer Nature Author services.

2) Please ensure that your manuscript meets PLOS ONE's style requirements, including those for file naming.

The manuscript has been adopted to the PLOS ONE Stlye requirements.

3) Thank you for including your ethics statement: 'All experiments were performed in compliance with the Institutional Review Board for the care of animals in accordance with the National Institutes of Health guidelines for ethical animal research (Ref. Nr. 113/14)'.

(a)Please amend your current ethics statement to confirm that your named ethics committee specifically approved this study.

(b) Once you have amended this/these statement(s) in the Methods section of the manuscript, please add the same text to the “Ethics Statement” field of the submission form (via “Edit Submission”)

The ethical approvement has been amended in the Method section, and as well in the Ethic Statement section.

4) The text that needs to be addressed involves the Results section (lines 320).

 In your revision please ensure you cite all your sources (including your own works), and quote or rephrase any duplicated text outside the methods section. Further consideration is dependent on these concerns being addressed.

We added the citation to this text. Hence, a special technique to evaluate the histology is explained, we did not rephrased it.

5) At this time, we ask that you please provide scale bars on the microscopy images presented in Figure 4 and refer to the scale bar in the corresponding Figure legend.

We added scale bars to Figure 4.

6) Please include the updated Competing Interests Statement and Funding Statement in your cover letter. We will change the online submission form on your behalf.

We updated the Funding Statement in the Cover letter. Thank you for your advice.

7)Your ethics statement must appear in the Methods section of your manuscript. If your ethics statement is written in any section besides the Methods, please move it to the Methods section and delete it from any other section. Please also ensure that your ethics statement is included in your manuscript, as the ethics section of your online submission will not be published alongside your manuscript.

The Ethic statement has been added to the method section, and canceled at the end of the manuscript.
